# Advanced techniques improve the performance of myocardial perfusion imaging

**DOI:** 10.1186/1532-429X-14-S1-P12

**Published:** 2012-02-01

**Authors:** Geraint Morton, Masaki Ishida, Andreas Schuster, Tobias Schaeffter, Amedeo Chiribiri, Eike Nagel

**Affiliations:** 1 King's College London, London, UK

## Background

Technical advances in cardiac magnetic resonance (CMR) perfusion imaging, particularly accelerated data acquisition methods, allow myocardial perfusion imaging with unprecedented spatio-temporal resolution. However, it is not clear how implementation of these advances affects perfusion image quality and artefacts and signal and contrast to noise ratios (SNR and CNR).

## Methods

A standard ultrafast gradient echo perfusion sequence (st-GrE) was compared with an advanced kt-accelerated steady state free precession sequence (kt-SSFP) at 1.5T in a hardware perfusion phantom, healthy volunteers (n=16) and patients (n=31) with known or suspected coronary artery disease. Volunteers had both sequences at rest in alternating order. Patients underwent stress imaging with either st-GrE (15) or kt-SSFP (16) prior to X-ray coronary angiography.

The phantom was used to generate signal intensity curves and noise maps for SNR and CNR analysis. Human images were analysed by a blinded observer using a nominal scale for quality (0 non-diagnostic, 1 poor, 2 moderate, 3 good, 4 excellent) and respiratory artefacts (0 non-diagnostic, 1 severe, 2 moderate, 3 minor, 4 nil) and also for the presence of CAD in patients. Other analyses included the extent (% affected segments), transmurality (1: 1-25%, 2: 26-50%, 3: 51-75%, 4 76-100%) and duration (frames) of dark rim artefacts (DRA). Segmental SNR and CNR were also quantified using the mid ventricular slice in volunteers.

## Results

Phantom studies demonstrated comparable SNR and CNR for both sequences: (SNR 54.7 & 53.9 and CNR 24.1 & 22.8 for st-GrE and kt-SSFP respectively). In normal hearts kt-SSFP imaging resulted in significantly improved image quality (p=0.003), SNR (21.0±6.7 vs. 18.8±6.6; p=0.009), CNR (15.4±6.1 vs. 14.0±6.0; p=0.034) and a reduced extent (p=<0.0001) and transmurality (p=0.0001) of DRA. In patients kt-SSFP imaging resulted in significantly improved image quality (p=0.012), and a reduced extent (p=<0.0001), duration (p=0.004) and transmurality (p=<0.0001) of DRA. Sensitivity and specificity for the detection of CAD against X-ray angiography was comparable with both sequences. There was a non-significant trend towards increased respiratory artefacts with kt-SSFP in both patients and volunteers.

## Conclusions

Advanced high spatio-temporal resolution CMR perfusion imaging using a kt-SSFP technique results in significantly improved image quality, SNR and CNR and a reduction in the extent and transmurality of DRA compared to a standard sequence.

## Funding

This work was supported by a European Union Grant (Grant number 224495 to GM, EN); the British Heart Foundation (Research Excellence Award RE/08/003 and FS/10/029/28253 to AS, EN); the Biomedical Research Centre (grant number BRC-CTF 196 to AS, EN) and the Wellcome Trust and EPSRC (grant number WT 088641/Z/09/Z to AC, EN).

**Table 1:** Perfusion sequence parameters

Parameter	st-GrE	kt-SSFP
Acquired spatial resolution	1.7 x 1.9 x 10mm	2.6 x 2.8 x 10mm
Echo time (TE)	Shortest (range 1.61-1.91ms)	Shortest (range 1.29-1.59ms)
Repetition time (TR)	Shortest (range 3.6-3.9ms)	Shortest (range 2.59-3.18ms)
Flip angle	18°	50°
Prepulse	90°	90°
Prepulse delay	100ms	100ms
Acceleration technique	SENSE: factor 2	kt-BLAST: factor 5 with 11 training profiles (effective k-t factor of 3.8)

**Table 2:** Sequence comparison

	Volunteers					Patients				
	**st-GRE**		**kt-SSFP**		**p value**	**st-GRE**		**kt-SSFP**		**p value**

**Image quality**	n	%	n	%		n	%	n	%	

**0 non-diagnostic**	1	6%	0			0		0		
**1 poor**	0		0			3	10%	0		
**2 moderate**	12	75%	3	19%	0.003	12	40%	2	6%	0.012
**3 good**	3	19%	12	75%		12	40%	17	53%	
**4 excellent**	0		1	6%		3	10%	13	41%	

**Respiratory artefacts**										

**0 non-diagnostic**	0		0			0		0		
**1 severe**	0		1	6%		0		2	6%	
**2 moderate**	3	19%	5	31%	0.07	7	23%	7	22%	0.17
**3 mild**	7	44%	7	44%		8	27%	16	50%	
**4 nil**	6	38%	3	19%		15	50%	7	22%	

**Dark rim artefact**										

**Extent(%)**	39±13		15±12		<0.0001	33±14		12±10		<0.0001
**Transmurality**	1.83±0.58		1.02±0.06		<0.0001	1.57±0.40		1.07±0.17		<0.0001
**Duration**	10.6±3.56		9.7±2.51		0.373	10.8±3.7		8.0±2.7		0.004

